# A Nomogram Predicting Overall and Cancer-Specific Survival of Patients with Primary Bone Lymphoma: A Large Population-Based Study

**DOI:** 10.1155/2020/4235939

**Published:** 2020-08-19

**Authors:** He-Hui Wang, Ke-Na Dai, A-Bing Li

**Affiliations:** ^1^Department of Orthopedics, Ningbo Yinzhou Second Hospital, Ningbo, 315100 Zhejiang, China; ^2^Department of Paediatrics, Ningbo Medical Center Lihuili Hospital, Ningbo, 315040 Zhejiang, China

## Abstract

We aimed to develop a nomogram for evaluating the overall survival (OS) and cancer-specific survival (CSS) in patients with primary bone lymphoma (PBL). Patients diagnosed with PBL between 2007 and 2016 were collected from the Surveillance, Epidemiology, and End Results (SEER) database. All patients were randomly allocated to the training cohort and validation cohort (2 : 1). The nomogram was developed by the training cohort and validated by the validation cohort using the concordance index (C-index), calibration plots, and decision curve analyses (DCAs). The C-index for CSS and OS prediction in the training cohort were 0.76 and 0.77, respectively; in the validation cohort, they were 0.76 and 0.79, respectively. The calibration curve showed good consistency between nomogram prediction and actual survival. The DCA indicated obvious net benefits of the new predictive model. The nomogram showed favorable applicability and accuracy, and it will be a reliable tool for predicting OS and CSS in patients with PBL.

## 1. Introduction

In 1928, Oberling first described an uncommon disease as primary lymphoma of bone. Subsequently, Parker and Jackson reported that this tumor was actually a malignant lymphoid infiltrating in bone [1, 2]. The most commonly used method for classifying PBL is the World Health Organization Classification of bone lymphoma, describing it as a malignant, lymphoid infiltrate within the bone, with or without cortical invasion or soft tissue extension, and without concurrent nodal or visceral involvement [3]. Traditionally, routine treatment has been radiation therapy, with a 5-year overall survival of approximately 45% as a single modality in patients with PBL [4]. Although the radiotherapy improves local outcomes, long-term failure to the treatment occurs in 50% [5]. Therefore, local radiotherapy is not sufficient for the treatment of PBL, even if it may be defined as a local disease. The introduction of multiagent chemotherapy in addition to radiotherapy has also improved the survival [6]. With the development of effective oncological treatments, surgery no longer played an important role in the management of PBL, apart from use in diagnosing isolated primary lymphoma of bone [7]. Previous studies suggested several clinical characteristics and prognostic factors for the prognosis in patients with PBL, including age, gender, race, tumor site, tumor stage, and use of radiotherapy and chemotherapy [5, 7–11]. However, these variables only served as single indexes and cannot precisely predict the survival in patients with PBL. In order to overcome the limitation of a single prognostic factor, a novel nomogram prognostic model is needed. Nomography is the graphical representation of mathematical relationships or laws that visualize the Cox regression models in a highly descriptive manner [12]. Nomograms are widely used as prognostic tools in oncology and medicine [13]. The SEER database of the National Cancer Institute, which covers approximately 30% of the US population [14], is an excellent resource for studying rare malignancies [15]. Therefore, we extracted records from the SEER database to determine the risk variables associated with OS and CSS in patients with PBL and to establish a nomogram to predict the prognosis in this population.

## 2. Materials and Methods

### 2.1. Patient Selection

The SEER database is a free cancer database available for public use. The population basis of the SEER database, which covers 30% of the entire US population and standardizes both classification and outcome criteria, is crucial, because it avoids selection biases and collects adequate numbers of cases for the study [16]. Retrospective analysis of PBL patients in the SEER database from 2007 to 2016 was performed. A total of 793 patients from 18 SEER registries were initially screened, using SEER∗Stat software (version 8.3.6; NCI; Bethesda, MD). The *International Classification of Diseases for Oncology* 3^rd^ edition was used to identify PBL based on site codes (C400-C41.9) and histology codes (9590/3, 9591/3, 9670/3, 19671/3, 9675/3, 9680/3, and 9684/3). Exclusion criteria were as follows [1]: unknown survival time [2]; missing or incomplete clinicopathological information (tumor stage, tumor location, race, and ethnicity) [3]; multiple primary cancers; and [4] patients under the age of 16.

### 2.2. Prognostic Variables

**Figure 1:**
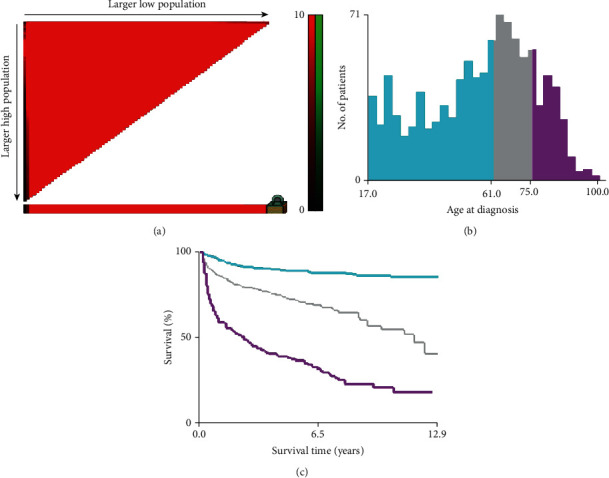
The graphs show the optimal cutoff values of age via X-tile analysis. (a) The black dot indicates that optimal cutoff values of age have been identified. (b) A histogram and (c) Kaplan-Meier were constructed based on the identified cutoff values. Optimal cutoff values of age were identified as 61 years and 75 years on survival.

Several clinicopathological variables, including age, gender, ethnicity, race, SEER tumor stage, tumor site, histologic subtype, treatments, and survival time, were examined. The tumor site was categorized as the axial skeleton (including the pelvis, vertebra, ribs, sternum, clavicle, and associated joints) and the appendicular skeleton (including the long and short bones of limbs and associated joints and scapula). The age of patients was stratified into three groups (<61, 61-75, or >75), and the cutoff points were determined using the X-tile program (Yale University, New Haven, CT, USA), which has been previously proven to determine optimal cutoff points of tumor variables [17] (Figure 1). The SEER stage was categorized as localized, regional, and distant using the SEER Program Coding and Staging Manual [18].

### 2.3. Construction and Validation of the Nomogram

All patients were randomly allocated in a 2 : 1 manner into the training and validation cohorts. The nomogram was developed based on the independent predictors which were included in the Cox regression models in the training cohort, and the nomogram was validated based on the validation cohort. The nomogram was built as described below. First, the univariate analysis was performed. Second, factors significantly correlated with survival (*p* < 0.2) in univariate analysis were included in a multivariate Cox regression analysis. A backward model selection was used to obtain the final multivariate model, with *p* values of <0.05 considered significant. The performance of the nomogram was assessed by the concordance index (C-index) and the calibration curve. The C-index has a range from 0.5 to 1.0, with 0.5 indicating random chance and 1.0 indicating perfect discrimination. The calibration curves were created to determine whether the predicted survival and actual survival were in concordance. DCA has been considered a novel approach that estimates predictive models from the clinical consequences by calculating the net benefits. Statistical software R (version 3.43, http://www.r-project.org) was used for all data analysis.

## 3. Results

### 3.1. Patient Characteristics

According to the inclusion and exclusion criteria, a total of 793 eligible PBL patients were identified from the SEER database. Demographic and clinical characteristics are listed in Table 1. Of the total, 529 eligible patients were allocated to the validation cohort and 264 eligible patients were allocated to the training cohort. Of these patients, 438 were male and 355 were female. The most common tumor location was the axial skeleton (438; 55.2%), and the major histology subtype was diffuse large B-cell (85.8%). The majority of patients were white (87.0%). Most patients were localized stage (443; 55.9%), 15.0% patients were regional stage, and 29.1% patients were distant stage. Most patients had received chemotherapy (82.7%) and radiotherapy (56.1%).

### 3.2. Nomogram Construction and Validation

**Figure 2:**
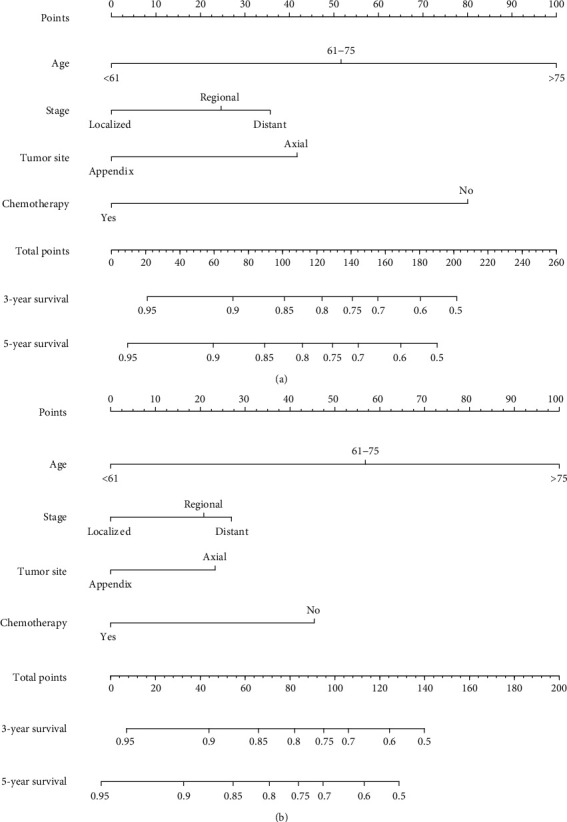
Nomograms for predicting the 3- and 5-year cancer-specific survival (a) and overall survival (b) of classical primary bone lymphoma patients. Description using nomograms: first, each feature point of the patient is assigned by plotting a vertical line to a point scale from the variable; then, sum all the points and draw a vertical line from the total point scale to the liver metastasis axis to obtain the probability.

**Figure 3:**
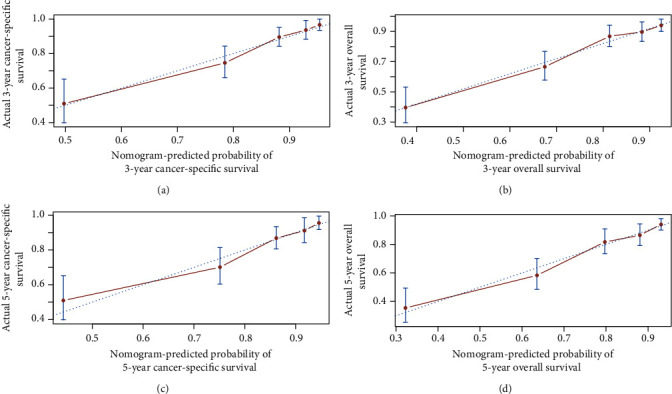
Training cohort calibration plot. (a) 3-year and (b) 5-year cancer-specific survival nomogram calibration curves. (c) 3-year and (d) 5-year overall survival nomogram calibration curves. The 45-degree line represents an ideal match between actual survival (*y*-axis) and nomogram-predicted survival (*x*-axis). The perpendicular line means 95% confidence intervals.

**Figure 4:**
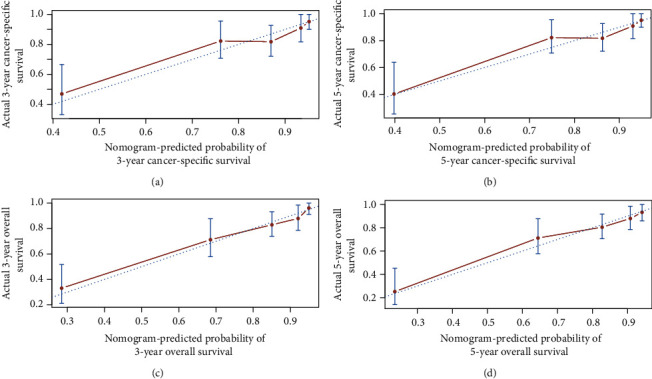
Validation cohort calibration plot. (a) 3-year and (b) 5-year cancer-specific survival nomogram calibration curves. (c) 3-year and (d) 5-year overall survival nomogram calibration curves. The 45-degree line represents an ideal match between actual survival (*y*-axis) and nomogram-predicted survival (*x*-axis). The perpendicular line means 95% confidence intervals.

**Figure 5:**
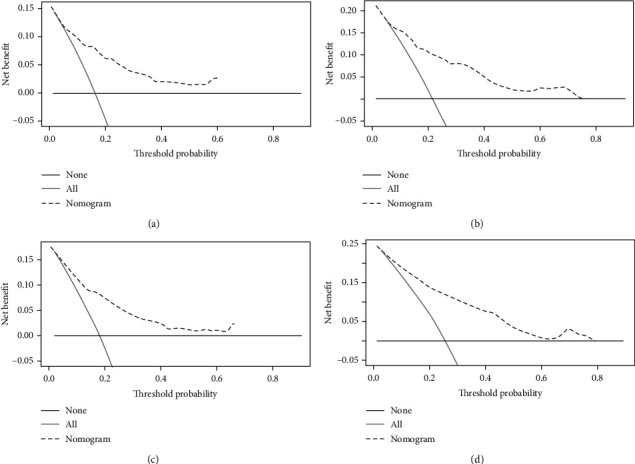
Decision curves of the nomogram predicting CSS at (a) 3 years and (b) 5 years and OS at (c) 3 years and (d) 5 years. The *x*-axis represents the threshold probabilities, and the *y*-axis measures the net benefit calculated by adding the true positives and subtracting the false positives. The horizontal line along the *x*-axis assumes that cancer-specific death occurred in no patients, whereas the solid gray line assumes that all patients will have cancer-specific death at a specific threshold probability. The dashed line represents the net benefit of using the nomogram. All = assume all patients survive. None = assume none survives.

Based on the training cohort, age, gender, ethnicity, race, tumor stage, tumor site, histologic subtype, surgery, chemotherapy, and radiotherapy were analyzed. Univariate analyses revealed that age, gender, tumor site, tumor stage, and chemotherapy were independent predictive factors for OS and CSS. Age, tumor stage, tumor site, and use of chemotherapy were identified as independent prognostic factors for OS and CSS after adjusting for other risk factors, whereas gender lost significance in the multivariate analysis (Tables 2 and 3). Nomograms that integrated all independent factors based on the multivariate models were constructed to predict 3- and 5-year CSS and OS in the training set (Figure 2). The nomogram gave every prognostic variable a score on the point scale (Table 4). By adding all the scores and locating total scores on the total scale, the estimated probability of 3- and 5-year CSS and OS of the individual patient can be identified. In the training cohort, the C-index of CSS and OS was 0.76 (95% CI, 0.75-0.81) and 0.77 (95% CI, 0.74-0.8), respectively. Likewise, in the validation cohort, the C-index was 0.76 (95% CI, 0.7-0.82) and 0.79 (95% CI, 0.69-0.81), respectively. The calibration plots of OS and CSS nomograms showed satisfactory agreement between nomogram prediction and actual observation (Figures 3 and 4). Based on the DCA, the nomogram showed great positive net benefits among almost all of the threshold probabilities at different time points, indicating the potential favorable clinical effect of the predictive model (Figure 5). This shows that the novel nomogram has high clinical practicability.

## 4. Discussion

Nomograms are reliable and convenient tools for estimating tumor prognosis [12, 19]. In the current study, a total of 793 PBL patients based on the SEER database were analyzed According to the multivariate analysis, the age, tumor site, tumor stage, and use of chemotherapy were independent prognostic factors for CSS and OS in patients with PBL. To the best of our knowledge, a nomogram applicable to PBL has not been established to date. This is the first study to develop and verify a nomogram for estimating the CSS and OS in patients with PBL based on the SEER database. The results of DCA indicated that the large net benefits of the nomogram can accurately predict CSS and OS.

Age is a significant predictor for the survival of malignant tumors in several studies [7, 11, 20]. Jawad et al. reported that PBL patients older than 60 years had worse survival outcomes [21], which is consistent with our results. Our analysis showed that compared with patients under the age of 61, patients between 61 and 75 and patients older than 75 years old have a higher risk of death for CSS and OS with 2 to 7 times.

In our study, most tumors appear within the axial skeleton, with approximately 55.2% occurring in the sites including pelvic bones, vertebrae, ribs, sternums, clavicles, bones of the skull and face, associated joints, and the mandible. This is inconsistent with the findings of previous studies [22–24]. A possible reason may be the different definitions of the location of tumors. We found that axial skeleton involvement was a significant variable correlated to worse survival with OS and CCS in the multivariable analysis when compared with the appendicular location. Demircay et al. reported that PBL patients with the involvement of pelvic bones, the spine, and jaws had poorer prognosis and decreased survival [25]. We found that the disease with the localized stage had a better prognosis compared with the regional or distant stage, which is in line with previous findings [21, 26]. Patients with only localized disease have been reported to have better survival [21], which can be identified as a unique clinical subgroup and might be suitable for local treatment strategies such as radiation, chemotherapy, and/or surgery.

With the development of oncological treatments, surgery is no longer a regular treatment in the management of PBL, except for the use in the initial biopsy to establish the diagnosis and the treat of pathological fractures. Therefore, surgery was not included as an analysis factor in this study. Some studies reported that combined modality treatment was associated with a better outcome than chemotherapy [27, 28] or radiotherapy alone [29, 30]. However, other studies indicated that no statistically significant difference was found in clinical efficacy between combined modality treatment and chemotherapy or radiotherapy alone [31, 32]. Christie et al. suggested that radiotherapy was the main method of securing local control and should remain a component of treatment [28]. In addition, Messina et al. found that radiotherapy was associated with better overall survival in diffuse large B-cell lymphoma [33]. But our study found that radiotherapy was not an independent predictive factor based on the univariate analysis in patients with PBL. This may due to the relatively common distant failure of radiotherapy, with a relapse rate of 50% [10]. Zinzani et al. concluded that the use of chemotherapy appeared to be more effective than radiotherapy alone based on an analysis of 52 consecutive patients with PBL [34]. Suryanarayan et al. also reported that nearly 90% of patients with early PBL were cured by chemotherapeutic drugs [35]. In our study, we found that the use of chemotherapy was an independent risk factor for survival, but the use of radiotherapy was not an independent prognostic factor. Due to the unknown information of the radiotherapy and chemotherapy regimen, subgroup analysis cannot be performed. Our findings may be biased, and this source was inherent in the SEER database. Therefore, these results should be interpreted with caution.

By integrating these independent prognostic factors, we built prognostic nomograms as statistical tools that can establish an effective prediction model to estimate 3- and 5-year OS and CSS for patients with PBL. In addition, nomograms are particularly suitable for assisting clinicians to assess individual survival probability at certain time intervals. For example, for a 70-year-old man, he was diagnosed with large B-cell PBL with a primary tumor in the femur. He then was diagnosed lung metastasis and received chemotherapy. Totaling the points of each prognostic predictor, he got 86.37 and 77.96 points in OS and CSS nomograms, respectively. According to the nomograms, 5-year CSS and OS were estimated to be 72% and 77%, respectively.

There are several limitations to our study. First, since the SEER database is a retrospective cohort, there is inevitably missing data that leads to reduced sample size. Prospective studies should be performed to further confirm our conclusion. Second, due to the limited duration of this retrospective study, we only evaluated 3- and 5-year survival as the primary endpoints. Considering the changes in treatment methods in different periods, we only extracted data from 2007 to 2016. Third, some variables associated with prognoses, such as fracture, multifocal disease, and use of rituximab, local recurrence, and detailed regimen of radiotherapy and chemotherapy, are unavailable in the SEER dataset. These variables may be an effective complement to this study, which will be an important section of our future research. Despite these shortcomings, the SEER database serves as an unparalleled resource when studying rare cancers. Finally, although the nomogram is developed based on a large cohort with validation, the predictive model should be validated in another database. Despite these limitations, we identified independent prognostic factors for survival for rare PBL that can assist clinicians in the assessment of the risk of survival with clinical significance.

## 5. Conclusion

Based on the SEER database, age, stage, tumor site, and chemotherapy are independent prognostic factors for both OS and CSS in patients with PBL. Our study is the first research to develop and validate a prognostic nomogram based on these variables. The nomogram showed favorable applicability and accuracy, and it will be a reliable tool for predicting OS and CSS in patients with PBL.

## Figures and Tables

**Table 1:** Patient demographics and pathological characteristics.

Characteristics	Training cohort *n* (%)	Validation cohort *n* (%)	Total *n* (%)
Age			
<61	257 (48.6)	117 (44.3)	374 (47.2)
61-75	167 (31.6)	91 (34.5)	258 (32.5)
>75	105 (19.8)	56 (21.2)	161 (20.3)
Gender			
Female	295 (55.8)	143 (54.2)	438 (55.2)
Male	234 (44.2)	121 (45.8)	355 (44.8)
Race			
White	456 (86.2)	234 (88.6)	690 (87.0)
Black	43 (8.1)	13 (4.9)	56 (7.1)
Other	30 (5.7)	17 (6.4)	47 (5.9)
Ethnicity			
Non-Spanish-Hispanic-Latino	454 (85.8)	238 (89.8)	691 (87.1)
Spanish-Hispanic-Latino	75 (14.2)	27 (10.2)	102 (12.9)
Tumor site			
Axial	284 (53.7)	154 (58.3)	438 (55.2)
Appendix	245 (46.3)	110 (41.7)	355 (44.8)
Stage			
Localized	296 (56.0)	147 (55.7)	443 (55.9)
Regional	74 (14.0)	45 (17.0)	119 (15.0)
Distant	159 (30.1)	72 (27.3)	231 (29.1)
Histology			
Diffuse large B-cell lymphoma	454 (85.8)	220 (83.3)	674 (85.0)
Malignant lymphoma	9 (1.7)	5 (1.9)	14 (1.8)
Non-Hodgkin lymphoma	62 (11.7)	35 (13.3)	97 (12.2)
Malignant lymphoma, small B lymphocytic	1 (0.2)	1 (0.4)	2 (0.3)
Lymphoplasmacytic lymphoma	2 (0.4)	3 (1.1)	5 (0.6)
Malignant lymphoma, large B, diffuse	1 (0.2)	0 (0.0)	1 (0.1)
Chemotherapy			
No	88 (16.6)	49 (18.6)	137 (17.3)
Yes	441 (83.4)	215 (81.4)	656 (82.7)
Radiation			
No	251 (47.4)	97 (36.7)	348 (43.9)
Yes	278 (52.6)	167 (63.3)	445 (56.1)

**Table 2:** Univariate and multivariate analyses of cancer-specific survival in the training cohort.

Characteristics	Univariate analysis	Multivariate analysis	*p*
	*p*	HR (95% CI)	
Age	<0.01		
<61		Reference group	
61-75		2.11 (1.16-3.84)	0.014
>75		3.93 (2.15-7.18)	<0.001
Gender	0.009		
Female		Reference group	
Male		1.36 (0.87-2.13)	0.178
Race	0.241		
White			
Black			
Other			
Ethnicity	0.561		
Non-Spanish-Hispanic-Latino			
Spanish-Hispanic-Latino			
Tumor site	<0.001		
Axial		Reference group	
Appendix		0.55 (0.34-0.89)	0.017
Stage	0.139		
Localized		Reference group	
Regional		1.42 (0.69-2.95)	0.346
Distant		1.66 (1.05-2.63)	0.032
Histology	0.491		
Diffuse large B-cell lymphoma			
Malignant lymphoma			
Non-Hodgkin lymphoma			
Malignant lymphoma, small B lymphocytic			
Lymphoplasmacytic lymphoma			
Malignant lymphoma, large B, diffuse			
Chemotherapy	<0.001		
No		Reference group	
Yes		0.33 (0.20-0.52)	<0.01
Radiation	0.489		
No			
Yes			

**Table 3:** Univariate and multivariate analyses of overall survival in the training cohort.

Characteristics	Univariate analysis	Multivariate analysis	*p*
	*p*	HR (95% CI)	
Age	<0.01		
<61		Reference group	
61-75		3.50 (1.82-5.12)	<0.001
>75		6.83 (4.09-11.41)	<0.001
Gender	0.009		
Female		Reference group	
Male		1.20 (0.84-1.72)	0.318
Race	0.683		
White			
Black			
Other			
Ethnicity	0.305		
Non-Spanish-Hispanic-Latino			
Spanish-Hispanic-Latino			
Tumor site	<0.001		
Axial		Reference group	
Appendix		0.63 (0. 43-0.93)	0.020
Stage	0.035		
Localized		Reference group	
Regional		1.54 (0.85-2.78)	0.153
Distant		1.69 (1.16-2.45)	0.006
Histology	0.259		
Diffuse large B-cell lymphoma			
Malignant lymphoma			
Non-Hodgkin lymphoma			
Malignant lymphoma, small B lymphocytic			
Lymphoplasmacytic lymphoma			
Malignant lymphoma, large B, diffuse			
Chemotherapy	<0.001		
No		Reference group	
Yes		0.42 (0.29-0.62)	<0.01
Radiation	0.742		
No			
Yes			

**Table 4:** Detailed scores of prognostic factors in the overall and cancer-specific survival nomograms.

Characteristics	OS nomogram	CSS nomogram
Age		
<61	0	0
61-75	50	50
>75	100	100
Tumor site		
Axial	41.75	24.08
Appendix	0	0
Stage		
Localized	0	0
Regional	18.18	13.99
Distant	36.37	27.96
Chemotherapy		
No	81.13	46.26
Yes	0	0

## Data Availability

Our data is available from the Surveillance, Epidemiology, and End Results (SEER) research database. This is a public research database.
